# Enhancing Muscle Strength and Reducing Inflammation: The Combined Effects of Hydrolyzed House Cricket Protein Supplementation and Concurrent Training in Older Women

**DOI:** 10.1002/fsn3.70984

**Published:** 2025-09-22

**Authors:** Pilanee Vaithanomsat, Udomlak Sukatta, Phornpimon Janchai, Piyaporn Tumnark, Jatuporn Phoemsapthawee

**Affiliations:** ^1^ Kasetsart Agricultural and Agro‐Industrial Product Improvement Institute Kasetsart University Bangkok Thailand; ^2^ Department of Sports Science, Faculty of Sports and Health Science Kasetsart University Nakhon Pathom Thailand

**Keywords:** anti‐inflammation, edible insect, exercise training, muscle strength, older adults, quality of life

## Abstract

This study examined the effects of hydrolyzed house cricket protein supplementation combined with concurrent training (CT) on muscle mass, strength, inflammation, and metabolic health in older women. In a 12‐week, double‐blind, randomized, placebo‐controlled trial, community‐dwelling older women (*n* = 39) were assigned to four groups: placebo (PLA), protein supplementation alone (PRO), CT with placebo (CT), or CT with protein supplementation (PRO+CT). Participants in the PRO and PRO+CT groups consumed 30 g of hydrolyzed cricket protein post‐exercise or at a matched time. The PRO+CT, PRO, and CT groups showed significant increases in fat‐free mass (*p* < 0.01 for PRO+CT and PRO; *p* < 0.05 for CT), with additional leg lean mass gains in the PRO+CT and PRO groups (both *p* < 0.05). Upper‐ and lower‐body strength improved in the PRO + CT (both *p* < 0.05) and CT groups, while the PRO group showed gains only in lower‐body strength (*p* < 0.05). Tumor necrosis factor‐alpha levels decreased in the PRO + CT and CT groups (*p* < 0.05 and *p* < 0.01), with insulin‐like growth factor‐1 significantly increased only in PRO+CT (*p* < 0.05). All groups improved 6 min walk test performance (*p* < 0.01 for PRO + CT and CT; *p* < 0.05 for PRO). Overall quality of life improved significantly in PRO+CT (*p* < 0.01) across physical, psychological, and social domains, with psychological gains also observed in CT. These findings suggest that combining hydrolyzed cricket protein supplementation with CT elicits synergistic benefits on muscle mass, strength, and functional capacity in older women, presenting a sustainable and effective strategy to counteract sarcopenia and support healthy aging.

## Graphical abstract

Combining hydrolyzed house cricket protein supplementation with concurrent training (CT) significantly enhanced muscle mass, strength, functional capacity, and quality of life in older women. The PRO+CT group showed superior improvements in fat‐free mass, strength, inflammatory markers, and insulin‐like growth factor‐1 compared to the placebo group. These results highlight the synergistic potential of insect protein and CT as a sustainable strategy to combat sarcopenia and promote healthy aging.

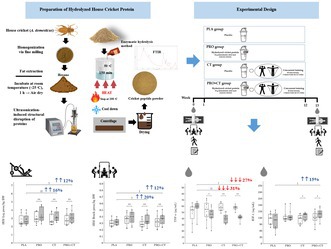

## 1 Introduction

Aging is accompanied by biological changes that progressively impair skeletal muscle function and cardiorespiratory fitness (CRF). Declines in CRF are hallmark indicators of physiological aging and are closely linked to heightened cardiometabolic risk. Moreover, reduced CRF is a strong predictor of hospitalization, morbidity, and mortality (Jackson et al. 2009). Concurrently, aging results in the gradual loss of muscle mass and strength—key contributors to functional decline and frailty (Cruz‐Jentoft et al. 2019). Individuals with functional impairments typically require more healthcare services, thereby increasing healthcare expenditures (DeJong et al. 2002). Notably, women are at greater risk of developing functional limitations than men. Although women typically enjoy greater longevity, they are also more likely to spend extended periods living with disability in later life (Newman and Brach 2001). As global populations age, the number of older women with functional decline is expected to rise substantially, posing a growing challenge to healthcare systems worldwide.

In addition to muscular function and CRF declines, aging is marked by significant changes in inflammatory and hormonal profiles that further contribute to functional deterioration. Chronic low‐grade inflammation is prevalent among older adults, reflected by elevated levels of inflammatory cytokines such as high‐sensitivity C‐reactive protein (hs‐CRP) and tumor necrosis factor‐alpha (TNF‐α) (Wang et al. 2017). Concurrently, aging is associated with reduced levels of anabolic hormones, particularly insulin‐like growth factor‐1 (IGF‐1), which is essential for preserving muscle mass and metabolic function (Bartke et al. 2003). The imbalance between increased inflammatory activity and diminished anabolic signaling is believed to underlie much of the age‐related decline in muscle and cardiometabolic health (Wang et al. 2017). Moreover, insulin resistance has been independently associated with reduced muscle mass and strength in non‐diabetic older adults, further compounding functional impairments (Seko et al. 2019; Yang et al. 2017).

To mitigate age‐related physiological decline, concurrent training (CT)—the structured combination of aerobic training (AT) and resistance training (RT)—is widely recognized as an effective intervention for enhancing both muscular strength and CRF in middle‐aged and older adults (Hurst et al. 2019; Khalafi et al. 2022; Markov et al. 2023; Schumann et al. 2022). This integrated modality is endorsed by current exercise guidelines, as it often yields superior outcomes compared to AT or RT alone. However, performing AT and RT within the same session may impose substantial physiological demands, leading to fatigue, energy depletion, and reduced training quality (El 2013; Fyfe et al. 2014; Knuiman et al. 2015). These factors can contribute to the so‐called interference effect, wherein CT attenuates the specific adaptations typically associated with each exercise mode (Coffey and Hawley 2017; Fyfe et al. 2014; Knuiman et al. 2015; Wilson et al. 2012).

To attenuate these interference effects, targeted nutritional strategies—particularly post‐exercise protein supplementation—have emerged as a promising adjunct (Perez‐Schindler et al. 2015). Consuming 20–40 g of protein after exercise has been shown to stimulate muscle protein synthesis, facilitate recovery, and support gains in muscular strength (Camera 2022; Camera et al. 2015). Moreover, adequate protein intake may enhance CRF by promoting mitochondrial biogenesis and capillary density (Knuiman et al. 2018). Nevertheless, most studies exploring protein supplementation in the context of CT have focused predominantly on younger or athletic populations, limiting the applicability of these findings to older adults (Alhebshi et al. 2021; Camera 2022; Camera et al. 2015; Forbes and Bell 2020; Jendricke et al. 2020; Knuiman et al. 2018; McAdam et al. 2018).

While CT has consistently been shown to improve muscular strength and CRF in adults aged 50 and older (Hurst et al. 2019; Khalafi et al. 2022; Markov et al. 2023), most studies on protein supplementation in this demographic have focused exclusively on RT, typically using whey, casein, essential amino acids (EAAs), or leucine (Deutz et al. 2014). Research examining the combined effects of CT and protein supplementation in older adults remains limited. Emerging evidence suggests that increased protein intake, whether through whole foods or leucine‐rich supplements, may further enhance muscle strength and physical function during multicomponent training (Gryson et al. 2014; Timmons et al. 2021). However, no studies to date have examined whether protein supplementation in conjunction with CT influences inflammatory markers or metabolic health in relation to muscle mass and strength in older adults. Addressing this gap could yield important insights into the synergistic potential of combining nutritional and exercise interventions to mitigate age‐related decline.

As interest in protein supplementation to support exercise adaptations in aging populations grows, the identification of sustainable and functionally effective protein sources becomes increasingly critical. Edible insects have emerged as a promising alternative, offering a sustainable and high‐quality source of nutrition. The house cricket ( *Acheta domesticus* ) is particularly notable for its rich nutritional profile—comprising 41.8%–75.2% protein, over 5% EAAs, and more than 500 mg of minerals—and for its environmentally efficient production (Giampieri et al. 2022; Ververis et al. 2022). Its high feed conversion efficiency, reproductive capacity, and low ecological footprint make it a compelling candidate for future food systems (Ververis et al. 2022). Cricket protein can be consumed directly or incorporated into various formulations. Recent interest has also focused on the development of bioactive peptides derived from cricket protein hydrolysates and their ability to retain biological activity under physiological conditions.

This study aimed to address these gaps by examining the effects of post‐exercise supplementation with 30 g of hydrolyzed house cricket protein, in combination with CT, on muscle mass, strength, inflammatory markers, and metabolic health related to muscle function in older women. We hypothesized that both hydrolyzed cricket protein supplementation and CT would independently improve these outcomes, with greater benefits observed when the two interventions were combined.

## 2 Materials and Methods

### 2.1 Study Design

**FIGURE 1:**
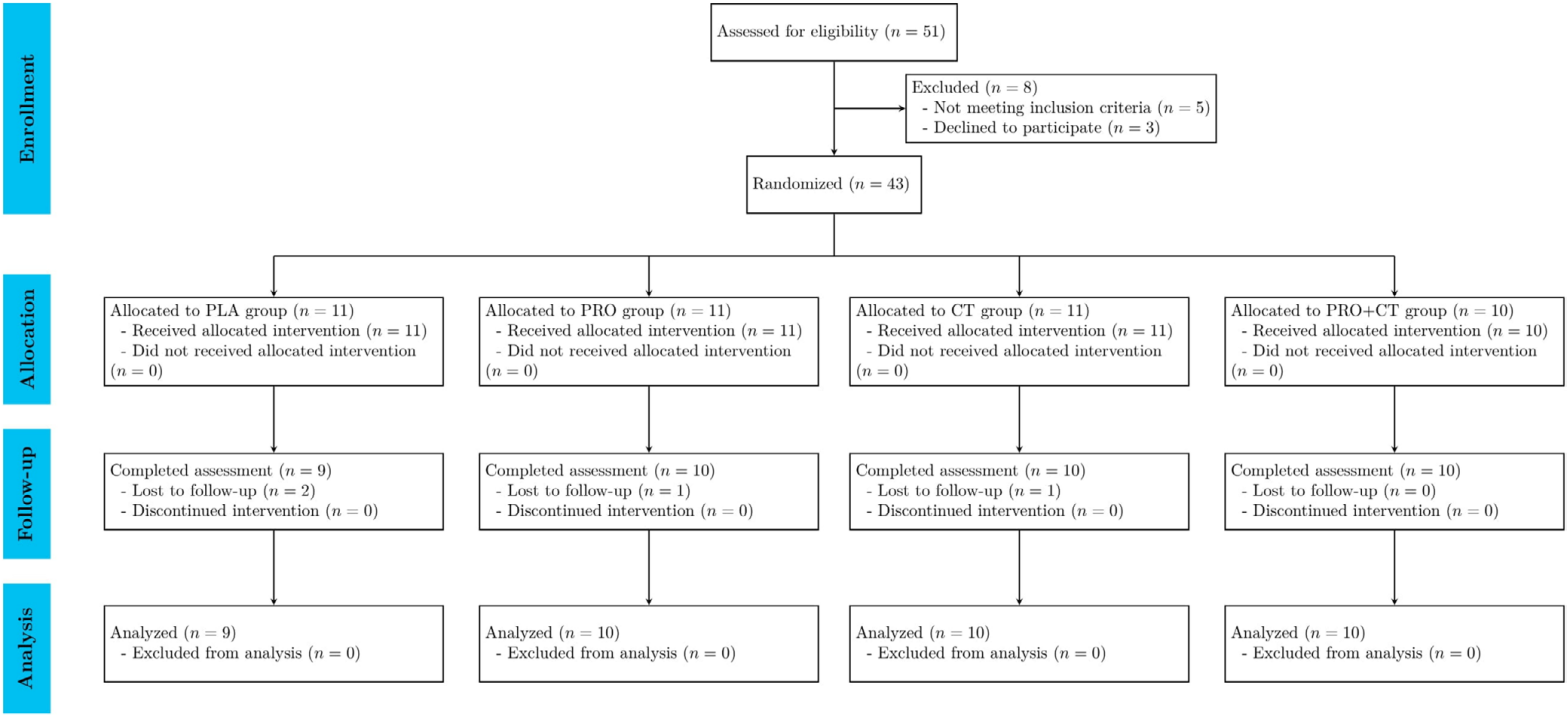
CONSORT flow diagram of the study.

A randomized, single‐blind, four‐arm controlled trial was conducted at the Faculty of Sports and Health Science, Kasetsart University, Thailand, between August 2021 and July 2022. Prior to enrollment, all participants were provided with comprehensive information about the study and gave written informed consent. The trial adhered to the Declaration of Helsinki and was approved by the Kasetsart University Research Ethics Committee (Approval No. COA64/042). The trial was also registered with the Thai Clinical Trials Registry (TCTR20230208010). Figure 1 illustrates the CONSORT flow diagram showing participant progression through each study phase. Following baseline assessments, participants were randomly allocated to one of four groups using the minimization method, stratified by age and lean mass (LM): (i) PLA group—no exercise intervention, receiving isocaloric placebo sachets; (ii) PRO group—no exercise intervention, receiving 30 g of hydrolyzed house cricket protein supplementation; (iii) CT group—CT, receiving isocaloric placebo sachets; and (iv) PRO+CT group—CT, receiving 30 g of hydrolyzed house cricket protein supplementation.

**FIGURE 2:**
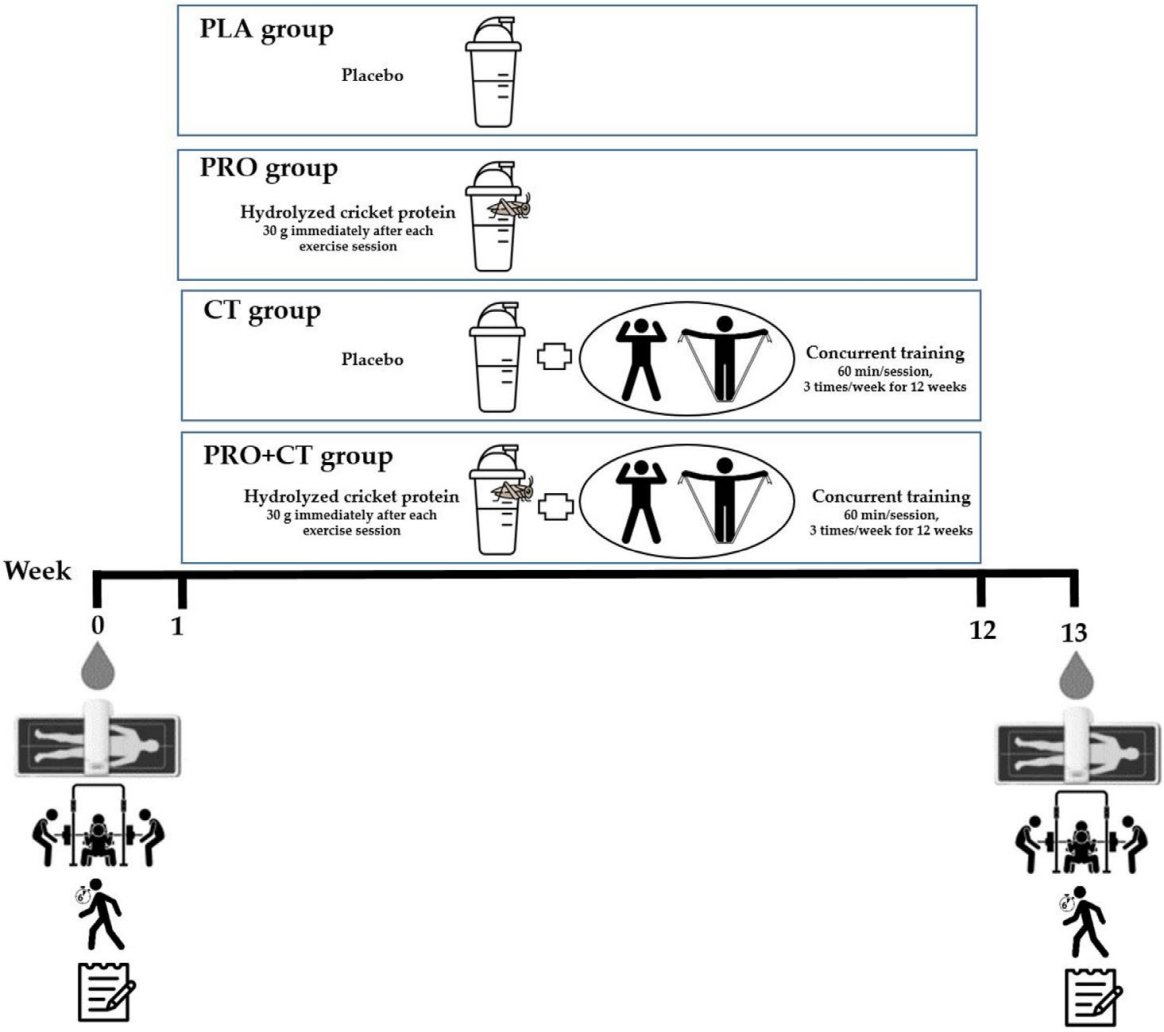
Overview of the experimental design.

Participants in the PLA group were instructed to maintain their usual physical activity and dietary patterns. Those in the CT and PRO+CT groups engaged in supervised group exercise sessions three times per week for 12 weeks, with each 60‐min session comprising RT and aerobic components. All participants were instructed to maintain their habitual dietary intake and lifestyle behaviors throughout the intervention. They were also asked to refrain from consuming any dietary supplements or vitamin products for the duration of the study. Dietary intake was monitored using a 3‐day dietary log (including two weekdays and 1 weekend day), which was analyzed to determine total energy and macronutrient intake. All outcome measures were assessed at baseline (approximately one week prior to the intervention) and again after the 12‐week intervention. To minimize diurnal variation, all assessments were conducted at the same time of day (±1 h) for each participant during both testing sessions. A schematic overview of the study protocol is illustrated in Figure 2.

### 2.2 Study Participants

A total of 51 community‐dwelling older women were initially screened through three Community Health Promotion Centers in Nakhon Pathom, Thailand. Of these, 43 met the eligibility criteria and provided written informed consent to participate in the study. Inclusion criteria were female sex; age ≥ 50 years (to reflect the early onset of sarcopenic changes (Peterson and Gordon 2011)); body mass index (BMI) between 18.5 and 30.0 kg/m^2^; non‐smoking status; independent ambulation without assistive devices; and a stable medication regimen for at least 3 months prior to enrollment. Participants were also required to demonstrate adequate cognitive function (Mini‐Mental State Examination score ≥ 24) and satisfactory nutritional status (Mini Nutritional Assessment–Short Form score ≥ 12). To reduce confounding from prior training adaptations, individuals who had engaged in structured RT or AT more than twice per week within the previous 6 months were excluded.

Exclusion criteria included a history of major cardiovascular or metabolic conditions (e.g., myocardial infarction, unstable cardiac disease, uncontrolled hypertension or diabetes, stroke, or other neurological disorders), ongoing cancer treatment, or hormone replacement therapy. Additional exclusions were musculoskeletal or orthopedic impairments limiting exercise (e.g., symptomatic osteoarthritis), recent acute illness or infection (within 2 weeks), severe psychiatric disorders, or known allergies to insect‐derived proteins. Participants with impaired hepatic or renal function, as evidenced by abnormal laboratory values, were also excluded.

### 2.3 Outcome Measurements

#### 2.3.1 Anthropometry and Body Composition

Standing height and body weight were measured using a stadiometer (Health o Meter Professional, Sunbeam Products Inc., USA) and a digital scale (Filizzola PL 150, Filizzola Ltd., Brazil), respectively, with participants barefoot and positioned according to the Frankfort horizontal plane. BMI was calculated as weight in kilograms divided by height in meters squared (kg/m^2^). Body composition was assessed in the morning following an overnight fast using dual‐energy X‐ray absorptiometry (DEXA) (Lunar iDXA, GE Healthcare, Chicago, IL, USA). Participants were scanned in a supine position with arms at their sides and feet supported to ensure standardized positioning. To minimize potential confounding from acute activity‐induced shifts in fluid or muscle mass, participants were instructed to avoid vigorous physical activity for 24 h prior to the assessment. The DEXA device was calibrated daily in accordance with the manufacturer's recommendations, and all scans were performed by trained technicians following standardized protocols. Body composition outcomes—including fat mass (FM), LM, and appendicular skeletal muscle mass (ASM)—were derived using enCORE software version 17.0 (GE Healthcare). The appendicular skeletal muscle index (ASMI) was calculated as ASM divided by height squared and also normalized by BMI.

#### 2.3.2 Upper and Lower Muscular Strength

The one‐repetition maximum (1RM) for both the leg press and bench press was estimated indirectly following the guidelines established by the National Strength and Conditioning Association. Prior to testing, participants completed a standardized warm‐up consisting of a single set of 10 repetitions using a light load that permitted 12–15 repetitions. During testing, if a participant was able to complete more than 10 repetitions with a given load, the resistance was increased by 30 pounds for the leg press or 10 pounds for the bench press. Each attempt was separated by a 3‐min rest interval. The estimated 1RM for each exercise was calculated using a standardized 1RM prediction chart based on the maximum weight lifted and the number of repetitions completed. Although movement cadence was not rigidly standardized, participants were instructed to perform each repetition in a controlled manner, emphasizing proper technique during both the eccentric and concentric phases. All tests were supervised by a certified strength and conditioning specialist to ensure safety and protocol adherence.

#### 2.3.3 Cardiorespiratory Fitness

Participants were instructed to wear appropriate exercise attire and to refrain from engaging in high‐intensity physical activity for at least 48 h prior to the assessment. CRF was assessed using the six‐minute walk test (6MWT), conducted along a 30‐m walkway in accordance with the guidelines of the American Thoracic Society (ATS) and the American College of Chest Physicians (ATS Committee on Proficiency Standards for Clinical Pulmonary Function Laboratories 2002). Participants were asked to walk as far as possible within 6 min without running. They were allowed to slow down, rest, or pause as needed, but were encouraged to resume walking as soon as they were able. While navigating the walkway, participants were required to turn quickly around cones at each end without hesitation. The total distance covered during the 6MWT was recorded, and estimated maximal oxygen consumption (V̇O_2_max) was calculated using a validated regression equation, as described by Šagát et al. (2023).

#### 2.3.4 Blood Samples and Analysis

All measurements were conducted following a 12‐h overnight fast at baseline and 72 h after the final training session to minimize acute exercise effects. Fasting blood samples were collected via venipuncture from the median cubital vein using standard commercial collection tubes. Samples were allowed to clot at room temperature for 20 min before serum separation by centrifugation at 1500 ***g*** for 10 min. Plasma samples were centrifuged under the same force for 15 min at 4°C. All specimens were subsequently aliquoted and stored at −80°C until analysis.

Serum IGF‐1 concentrations were measured using a commercial chemiluminescence immunoassay kit and analyzer (Shenzhen New Industries Biomedical Engineering Co. Ltd., Shenzhen, China). Plasma TNF‐α levels were quantified using an enzyme‐linked immunosorbent assay (Cayman Chemical Company, Ann Arbor, MI, USA), while serum hs‐CRP concentrations were determined via immunonephelometric assay (BioTécnica Indústria e Comércio Ltda., Minas Gerais, Brazil). Fasting blood glucose (FBG), total cholesterol (TC), triglycerides (TG), high‐density lipoprotein cholesterol (HDL‐c), and low‐density lipoprotein cholesterol (LDL‐c) were analyzed using an automated biochemical analyzer (Thermo Fisher Scientific Inc., Waltham, MA, USA).

To assess cardiovascular risk, atherogenic indices were calculated as follows: the atherogenic index of plasma (AIP) was defined as log_10_ (TG/HDL‐c), reflecting the balance between atherogenic and protective lipoproteins. Castelli Risk Index I (CRI‐I) and II (CRI‐II) were calculated as TC/HDL‐c and LDL‐c/HDL‐c, respectively. The atherogenic coefficient (AC) was determined as (TC − HDL‐c)/HDL‐c, indicating the proportion of non‐HDL cholesterol relative to HDL‐c. Serum creatine kinase (CK) activity was assessed using an enzymatic rate method (Beckman Coulter UniCel DxC 800 Chemistry Analyzer, MN, USA). Fasting insulin levels were measured with an immunoassay analyzer (Siemens Healthcare Diagnostics, Mannheim, Germany), and insulin resistance was estimated using the homeostasis model assessment of insulin resistance (HOMA‐IR), calculated as [fasting insulin (μIU/mL) × FBG (mmol/L)]/22.5 (Matthews et al. 1985). In addition, serum levels of aspartate transaminase (AST), alanine transaminase (ALT), blood urea nitrogen (BUN), and creatinine were analyzed using commercial assay kits with a clinical chemistry analyzer (BS‐360E, Mindray Bio‐Medical Electronics Co. Ltd., Shenzhen, China).

#### 2.3.5 Quality of Life

Quality of life (QoL) was assessed using the Thai version of the World Health Organization Quality of Life—Brief (WHOQOL‐BREF) questionnaire (WHOQOL Group 1994). This instrument comprises 26 items categorized into four domains: physical health (7 items), psychological health (6 items), social relationships (3 items), and environmental factors (8 items). Additionally, two global items assess overall QoL and general health status. Of the 26 items, 23 are positively worded, while 3 are negatively worded. All responses are rated on a 5‐point Likert scale. Positively phrased items are scored from “not at all” (1) to “extremely” (5), whereas negatively phrased items are reverse‐scored to maintain consistency in interpretation.

#### 2.3.6 Dietary Intake

To evaluate potential changes in dietary intake over the intervention period, participants completed a 3‐day food diary both prior to and following the 12‐week intervention. Dietary records included two weekdays and one weekend day and were documented using a standardized booklet provided by the research team. Nutrient intake data were analyzed for total energy and macronutrient composition using INMUCAL version 3.0 (Institute of Nutrition, Mahidol University, Nakhon Pathom, Thailand) (Banjong et al. 2016).

### 2.4 Concurrent Training Program

The CT program consisted of three supervised sessions per week, each lasting 60 min over a 12‐week period. Each session comprised a 10‐min dynamic warm‐up, 40 min of combined aerobic and resistance exercises, and a 10‐min cool‐down with static stretching. All sessions were conducted under the supervision of experienced sport scientists to ensure participant safety and adherence to the protocol. RT utilized body weight and elastic resistance bands, targeting all major muscle groups with progressively increasing intensity and volume. The program's content validity, evaluated by three experts in strength training, was rated as good (IOC = 0.87). Exercise intensity was monitored throughout using heart rate monitors (H10, Polar Electro Inc., Finland) to ensure consistent cardiovascular load and safety.

Each session began with 20 min of low‐impact aerobic exercises (e.g., side taps with punches, leg curls, knee‐ups, side‐to‐side rocking) performed at 70%–80% of heart rate reserve. This was followed by resistance exercises, including push‐ups, bent‐over rows, lateral raises, biceps curls, triceps extensions, squats, lunges, lateral walks, side bends, and planks. Participants completed two sets of 8–12 repetitions per exercise, with 30 s of active recovery between sets. All movements were executed through a full range of motion, and exercise progression was individualized based on participants' performance and perceived exertion using the OMNI‐Resistance Exercise Scale (range: 0 = extremely easy to 10 = extremely hard) (Lagally and Robertson 2006). Band resistance was increased (i.e., color changed) when participants were capable of performing at least two additional repetitions in the second set and reported a perceived exertion rating below 7 for the target muscle group. Although participants had prior RT experience, detailed instructions were provided before each session. Proper breathing techniques were reinforced, with an emphasis on exhaling during the concentric phase and inhaling during the eccentric phase, while maintaining a controlled, self‐paced rhythm throughout the exercises.

### 2.5 Preparation of Hydrolyzed House Cricket Protein Supplementation

The primary raw material used in this study was the house cricket ( *A. domesticus* ). The insects were finely milled into a homogeneous powder and stored in airtight plastic containers at −20°C to maintain quality prior to use. Proximate composition analyses—including moisture, ash, protein, lipid content, and microbiological quality—were performed according to standardized methods established by the Association of Official Analytical Chemists (AOAC 2000).

Cricket protein hydrolysate was prepared using a modified enzymatic hydrolysis method, adapted from previous studies (Hall and Liceaga 2020; Vaithanomsat and Punyasawon 2008). Briefly, dry ground cricket powder was mixed with water to achieve a protein concentration of 6.5% w/w, yielding a final mixture volume of 100 g. Enzymatic hydrolysis was performed using bromelain, a plant‐derived protease extracted from pineapple rhizomes (Insuan et al. 2021). The pH of the suspension was adjusted to the desired level using 4 N NaOH and maintained throughout the reaction. Hydrolysis was carried out at 50°C for 120 min with continuous stirring, using an enzyme concentration of 1.5% v/w. To terminate the reaction, the hydrolysate was heated to 100°C for 15 min. The resulting mixture was then cooled, and the pH of the supernatant was adjusted to 7.0 using 1 N HCl. The precipitate was removed by centrifugation at 8000 rpm for 15 min at 4°C. The clarified hydrolysate was spray‐dried to obtain the final cricket protein hydrolysate powder.

The final peptide‐rich powder exhibited the following proximate composition: protein (76.96%), carbohydrates (4.53%), fat (0.55%), dietary fiber (9.48%), ash (6.75%), and moisture (1.73%). Analysis of its free amino acid content revealed a total of 57.00 mg per 100 g of hydrolysate. Glutamic acid was the most abundant amino acid (9.20), followed by aspartic acid (7.17), alanine (5.08), and leucine (4.37 mg/100 g), as detailed in Table S1.

### 2.6 Statistical Analysis

All data are presented as means ± standard deviations (SD). Data normality was assessed using the Shapiro–Wilk test. When deviations from normality were detected, log transformations were applied to meet parametric assumptions. Baseline differences among groups were evaluated using one‐way analysis of variance (ANOVA) for continuous variables and the Chi‐square test for categorical variables. To examine the effects of hydrolyzed house cricket protein supplementation and CT over time, a two‐way repeated measures ANOVA was conducted with group (PLA, PRO, CT, PRO+CT) and time (baseline vs. 12 weeks) as factors. When significant group‐by‐time interactions or main effects were identified, univariate post hoc analyses were conducted to determine specific between‐ or within‐group differences. Effect sizes were calculated using eta‐squared (*η* ^*2*^) and interpreted as small (0.01), medium (0.06), or large (0.14). Additionally, analysis of covariance (ANCOVA) was used to compare between‐group differences at follow‐up, adjusting for baseline values to enhance the precision of treatment effect estimates. Statistical significance was set at *p* < 0.05. All analyses were performed using SPSS version 26.0 (IBM Corp., Armonk, NY, USA).

A priori power analysis was conducted using G*Power software (Version 3.1.9.2; Düsseldorf, Germany), based on effect size estimates from Holm et al. (2008), who examined muscle strength adaptations following 24 weeks of protein‐enriched supplementation combined with RT in postmenopausal women. To detect significant changes in relative strength using repeated measures ANOVA with one covariate, a minimum total sample size of 34 participants (approximately 9 per group) was required, assuming an alpha level of 0.05, statistical power (1–*β*) of 0.80, and a large effect size (Cohen's *f* = 0.60). To ensure adequate power while accounting for an anticipated 10% dropout rate, the final target sample size was set at 38 participants, or approximately 10 per group.

## 3 Results

A total of 39 older women completed the study, with a mean age of 64.7 ± 5.5 years. At baseline, participants had an average BMI of 23.7 ± 2.9 kg/m^2^, a height of 154.0 ± 4.3 cm, and fat‐free mass (FFM) of 35.0 ± 3.4 kg. Their estimated V̇O_2_max was 25.2 ± 3.2 mL/kg/min. Muscular strength was assessed using indirect 1RM testing, yielding average values of 159.8 ± 36.0 kg for the leg press and 17.6 ± 4.0 kg for the bench press. The baseline characteristics of participants are summarized in Table 1. No significant differences were observed between groups, indicating comparability in blood pressure, anthropometric measures, body composition, CRF, upper and lower body muscular strength, comorbidity prevalence, and medication use. Additionally, none of the participants exhibited malnutrition or cognitive impairment at baseline.

**TABLE 1:** Participants' characteristics in the study.

	Total (*n* = 39)	PLA (*n* = 9)	PRO (*n* = 10)	CT (*n* = 10)	PRO + CT (*n* = 10)	*p*
Age (years)	64.7 ± 5.5	64.3 ± 4.9	63.0 ± 5.5	66.0 ± 5.1	65.4 ± 6.7	0.651
HR (beats/min)	70.9 ± 8.5	72.8 ± 6.0	72.2 ± 10.0	71.4 ± 6.9	67.5 ± 10.4	0.527
SBP (mmHg)	126.6 ± 15.0	126.9 ± 16.9	124.6 ± 11.7	128.4 ± 13.9	126.6 ± 18.9	0.957
DBP (mmHg)	72.6 ± 7.3	73.6 ± 8.4	74.3 ± 6.7	73.0 ± 6.8	69.5 ± 7.4	0.481
MAP (mmHg)	90.6 ± 8.3	91.4 ± 9.8	91.1 ± 7.5	91.4 ± 9.0	88.6 ± 8.0	0.860
Height (cm)	154.0 ± 4.3	153.1 ± 5.6	153.5 ± 4.9	153.6 ± 3.0	155.8 ± 3.3	0.503
BW (kg)	56.1 ± 6.5	58.7 ± 5.7	53.7 ± 8.1	55.8 ± 5.0	56.5 ± 6.8	0.418
BMI (kg/m^2^)	23.7 ± 2.9	25.1 ± 2.7	22.8 ± 3.5	23.7 ± 2.5	23.3 ± 2.9	0.284
FM (kg)	21.0 ± 4.9	22.2 ± 4.4	18.9 ± 5.9	21.1 ± 3.7	21.7 ± 5.3	0.464
FFM (kg)	35.0 ± 3.4	35.8 ± 3.7	35.0 ± 3.9	34.9 ± 2.7	34.5 ± 3.5	0.884
Estimated V̇O_2_max (mL/kg/min)	25.2 ± 3.2	24.2 ± 3.2	26.5 ± 3.7	25.0 ± 2.4	24.9 ± 3.4	0.451
1RM Leg press (kg)	159.8 ± 36.0	159.2 ± 36.0	163.5 ± 37.1	156.6 ± 36.4	159.8 ± 39.8	0.966
1RM Bench press (kg)	17.6 ± 4.0	17.6 ± 3.1	18.8 ± 3.5	15.9 ± 3.5	17.9 ± 5.4	0.468
Comorbidities						
Hypertension (*n*, %)	20 (51.3%)	4 (44.4%)	5 (50.0%)	6 (60.0%)	5 (50.0%)	0.922
Type 2 diabetes (*n*, %)	4 (10.3%)	1 (11.1%)	1 (10.0%)	1 (10.0%)	1 (10.0%)	1.000
Dyslipidemia (*n*, %)	8 (20.5%)	2 (22.2%)	2 (20.0%)	2 (20.0%)	2 (20.0%)	0.999
Medication						
Antihypertensive drugs (*n*, %)	8 (20.5%)	2 (22.2%)	2 (20.0%)	2 (20.0%)	2 (20.0%)	0.999
Antihyperglycemic drugs (*n*, %)	4 (10.3%)	1 (11.1%)	1 (10.0%)	1 (10.0%)	1 (10.0%)	1.000
Statins (*n*, %)	8 (20.5%)	2 (22.2%)	2 (20.0%)	2 (20.0%)	2 (20.0%)	0.999
MMSE score	25.3 ± 1.1	25.3 ± 1.3	25.5 ± 1.1	25.2 ± 1.0	25.2 ± 0.9	0.916
MNA‐SF score	12.7 ± 0.7	12.7 ± 0.9	12.6 ± 0.7	12.7 ± 0.8	12.7 ± 0.6	0.990

*Note:* Data are presented as means ± SD and percentage.

Abbreviations: 1RM, one‐repetition maximum; BMI, body mass index; BW, body weight; CT, concurrent exercise training with placebo supplementation; DBP, diastolic blood pressure; FFM, fat‐free mass; FM, fat mass; HR, heart rate; MAP, mean arterial pressure; MMSE, Mini‐Mental State Examination; MNA‐SF, Mini Nutritional Assessment–Short Form; PLA, no exercise training with placebo supplementation; PRO, no exercise training with hydrolyzed house cricket protein supplementation; PRO+CT, concurrent exercise training with hydrolyzed house cricket protein supplementation; SBP, systolic blood pressure; V̇O_2_max, maximal oxygen uptake.

Throughout the intervention, average daily energy intake remained stable across all groups (Table S2). Protein intake averaged approximately 1.1–1.2 g/kg BW/day, contributing 14.2% of total daily energy intake, with no significant group differences. Habitual physical activity levels also remained unchanged during the study period. During the training phase, both the CT and PRO+CT groups exhibited full compliance with the prescribed exercise regimen, achieving 100% session attendance. The hydrolyzed house cricket protein supplement was well tolerated, with no adverse effects reported. Furthermore, no significant changes in liver or kidney enzyme levels were detected, supporting the safety and tolerability of the supplementation protocol.

### 3.1 Body Composition

A two‐way ANOVA with large effect sizes (*η* ^2^) revealed statistically significant group‐by‐time interactions for body composition variables, including FFM (*F*_(3,35)_ = 10.974, *p* = 0.000, *η* ^2^ = 0.485), leg lean LM (*F*_(3,35)_ = 4.263, *p* = 0.011, *η* ^2^ = 0.268), android FM (*F*_(3,35)_ = 4.475, *p* = 0.009, *η* ^2^ = 0.277), and android‐to‐gynoid (A/G) fat ratio (*F*_(3,35)_ = 5.573, *p* = 0.003, *η* ^2^ = 0.323). Additionally, significant main effects of time were observed for several outcomes, including body fat percentage (%BF) (*F*_(1,35)_ = 17.719, *p* = 0.000, *η* ^2^ = 0.336), FM (*F*_(1,35)_ = 12.301, *p* = 0.001, *η* ^2^ = 0.260), FFM (*F*_(1,35)_ = 82.075, *p* = 0.000, *η* ^2^ = 0.701), skeletal muscle mass (SMM) (*F*_(1,35)_ = 12.858, *p* = 0.001, *η* ^2^ = 0.269), arms LM (*F*_(1,35)_ = 8.000, *p* = 0.008, *η* ^2^ = 0.186), legs LM (*F*_(1,35)_ = 7.426, *p* = 0.010, *η* ^2^ = 0.175), trunk LM (*F*_(1,35)_ = 12.160, *p* = 0.001, *η* ^2^ = 0.258), height‐adjusted appendicular skeletal muscle index (ASMI^ht^) (*F*_(1,35)_ = 16.396, *p* = 0.000, *η* ^2^ = 0.319), BMI‐adjusted ASMI (ASMI^BMI^) (*F*_(1,35)_ = 7.018, *p* = 0.012, *η* ^2^ = 0.167), android FM (*F*_(1,35)_ = 21.963, *p* = 0.000, *η* ^2^ = 0.386), and A/G ratio (*F*_(1,35)_ = 29.668, *p* = 0.000, *η* ^2^ = 0.459).

Post hoc analyses revealed significant within‐group improvements following the 12‐week intervention. In the PRO group, participants exhibited significant reductions in FM (*p* < 0.05), android FM (*p* < 0.01), and A/G ratio (*p* < 0.01), along with significant increases in FFM (*p* < 0.01), leg LM (*p* < 0.01), and ASMI^ht^ (*p* < 0.01) compared to baseline. Relative to the PLA group, the PRO group demonstrated significantly lower android FM (*p* < 0.05) and A/G ratios (*p* < 0.01) and greater increases in FFM (*p* < 0.01) and leg LM (*p* < 0.05). In the CT group, significant reductions in %BF (*p* < 0.01), FM (*p* < 0.01), android FM (*p* < 0.01), and A/G ratio (*p* < 0.01) were observed, along with significant increases in FFM (*p* < 0.01), SMM (*p* < 0.05), arm LM (*p* < 0.05), and trunk LM (*p* < 0.01). When compared with the PLA group, the CT group showed significantly lower android FM (*p* < 0.05), reduced A/G ratio (*p* < 0.05), and greater gains in FFM (*p* < 0.05).

The PRO+CT group exhibited the most comprehensive improvements. After 12 weeks, this group showed significant reductions in %BF (*p* < 0.01), android FM (*p* < 0.01), and A/G ratio (*p* < 0.01), as well as significant increases in FFM (*p* < 0.01), SMM (*p* < 0.01), arm LM (*p* < 0.05), leg LM (*p* < 0.01), trunk LM (*p* < 0.05), ASMI^ht^ (*p* < 0.01), and ASMI_BMI (*p* < 0.05) compared to baseline. Furthermore, relative to the PLA group, the PRO+CT group had significantly lower android FM (*p* < 0.05) and A/G ratio (*p* < 0.05), along with greater increases in FFM (*p* < 0.01) and leg LM (*p* < 0.05). These findings are summarized in Table 2.

**TABLE 2:** Body composition parameters at baseline and after the 12‐week intervention.

	PLA (*n* = 9)		PRO (*n* = 10)		CT (*n* = 10)		PRO + CT (*n* = 10)		Time effect *η* ^2^ (*p*‐value)	Group × Time interaction *η* ^2^ (*p*‐value)
	Baseline	Post‐test	Baseline	Post‐test	Baseline	Post‐test	Baseline	Post‐test		
BW (kg)	58.7 ± 5.7	57.9 ± 7.3	53.7 ± 8.1	54.0 ± 7.8	55.8 ± 5.0	55.8 ± 5.1	56.5 ± 6.8	56.6 ± 6.3	0.003 (0.733)	0.068 (0.478)
BMI (kg/m^2^)	25.1 ± 2.7	24.7 ± 2.8	22.8 ± 3.5	23.0 ± 3.3	23.7 ± 2.5	23.7 ± 2.5	23.3 ± 2.9	23.3 ± 2.6	0.006 (0.653)	0.105 (0.269)
%BF (%)	39.3 ± 4.0	39.0 ± 4.0	35.6 ± 6.7	35.3 ± 6.6	38.4 ± 6.7	37.3 ± 4.3^a^**	39.3 ± 5.9	38.2 ± 5.4^a^**	0.336 (0.000)^††,^**	0.161 (0.101)
FM (kg)	22.2 ± 4.4	22.2 ± 4.5	18.9 ± 5.9	18.3 ± 5.8^a^*	21.1 ± 3.7	20.2 ± 4.1^a^**	21.7 ± 5.3	21.4 ± 5.2	0.260 (0.001)^††,^**	0.131 (0.173)
FFM (kg)	35.2 ± 2.9	35.2 ± 2.6	35.0 ± 3.9	36.0 ± 3.8^a^**^,b^**	34.9 ± 2.7	35.6 ± 2.8^a^**^,b^*	34.5 ± 3.5	35.7 ± 3.4^a^**^,b^**	0.701 (0.000)^††,^**	0.485 (0.000)^††,^**
SMM (kg)	33.4 ± 2.6	33.3 ± 2.1	33.2 ± 3.7	33.7 ± 3.7	33.1 ± 2.6	33.8 ± 2.8^a^*	32.7 ± 3.4	33.5 ± 3.2^a^**	0.269 (0.001)^††,^**	0.184 (0.066)
Arms LM (kg)	3.35 ± 0.5	3.32 ± 0.5	3.47 ± 0.4	3.50 ± 0.4	3.38 ± 0.5	3.48 ± 0.4^a^*	3.40 ± 0.4	3.50 ± 0.4^a^*	0.186 (0.008)^††,^**	0.172 (0.081)
Legs LM (kg)	11.3 ± 1.2	11.1 ± 1.1	11.0 ± 1.6	11.3 ± 1.7^a^**^,b^*	11.3 ± 1.1	11.4 ± 1.1	11.0 ± 1.1	11.2 ± 1.1^a^**^,b^*	0.175 (0.010)^††,^*	0.268 (0.011)^††,^*
Trunk LM (kg)	15.4 ± 1.7	15.4 ± 1.6	15.8 ± 1.9	15.9 ± 1.6	15.6 ± 1.3	16.1 ± 1.6^a^**	15.3 ± 1.9	15.7 ± 1.8^a^*	0.258 (0.000)^††,^**	0.154 (0.115)
ASMI^ht^	6.3 ± 0.4	6.3 ± 0.5	6.1 ± 0.9	6.3 ± 0.9^a^**	6.2 ± 0.7	6.3 ± 0.7	5.9 ± 0.6	6.1 ± 0.7^a^**	0.319 (0.012)^††,^*	0.194 (0.0154)
ASMI^BMI^	0.61 ± 0.1	0.61 ± 0.0	0.64 ± 0.1	0.65 ± 0.1	0.62 ± 0.1	0.63 ± 0.1	0.62 ± 0.1	0.64 ± 0.1^a^*	0.167 (0.012)^††,^*	0.025 (0.824)
Android FM (kg)	1.8 ± 0.6	1.9 ± 0.7	1.3 ± 0.7	1.2 ± 0.7^a^**^,b^*	1.6 ± 0.7	1.5 ± 0.7^a^**^,b^*	1.8 ± 0.6	1.6 ± 0.6^a^**^,b^*	0.386 (0.000)^††,^**	0.277 (0.009)^††,^**
Gyniod FM (kg)	3.6 ± 0.6	3.5 ± 0.6	3.2 ± 0.9	3.2 ± 0.9	3.4 ± 0.5	3.4 ± 0.5	3.6 ± 0.8	3.6 ± 0.9	0.008 (0.602)	0.128 (0.5181)
A/G ratio	0.52 ± 0.1	0.54 ± 0.1	0.43 ± 0.2	0.36 ± 0.2^a^**^,b^**	0.49 ± 0.2	0.44 ± 0.2^a^**^,b^*	0.50 ± 0.1	0.45 ± 0.1^a^**^,b^*	0.459 (0.000)^††,^**	0.323 (0.029)^††,^*

*Note:* Data are presented as mean ± SD. Superscript “††” indicates *η* ^2^ ≥ 0.14; * and ** indicate *p* < 0.05 and *p* < 0.01, respectively. Superscript “a” indicates significant differences from baseline within the group; “b” indicates significant differences compared to the PLA group at 12 weeks.

Abbreviations: %BF, body fat percentage; ASMI^ht^, appendicular skeletal muscle mass normalized by height squared; BMI, body mass index; BW, body weight; CT, concurrent exercise training with placebo supplementation; FFM, fat‐free mass; FM, fat mass; LM, lean mass; PLA, no exercise training with placebo supplementation; PRO, no exercise training with hydrolyzed house cricket protein supplementation; PRO+CT, concurrent exercise training with hydrolyzed house cricket protein supplementation; SMI^BMI^, appendicular skeletal muscle mass normalized by body mass index; SMM, skeletal muscle mass.

### 3.2 Upper and Lower Muscular Strength

A two‐way ANOVA with large *η* ^2^ revealed statistically significant group‐by‐time interactions for muscular strength outcomes. These included absolute leg press 1RM (*F*_(3,35)_ = 5.611, *p* = 0.003, *η* ^2^ = 0.325), relative leg press 1RM/kg BW (*F*_(3,35)_ = 4.761, *p* = 0.007, *η* ^2^ = 0.290), absolute bench press 1RM (*F*_(3,35)_ = 4.139, *p* = 0.013, *η* ^2^ = 0.262), and relative bench press 1RM/kg BW (*F*_(3,35)_ = 3.933, *p* = 0.016, *η* ^2^ = 0.252). In addition, main effects of time were statistically significant for all strength parameters, including absolute leg press 1RM (*F*_(1,35)_ = 56.292, *p* = 0.000, *η* ^2^ = 0.617), relative leg press 1RM/kg BW (*F*_(1,35)_ = 65.813, *p* = 0.000, *η* ^2^ = 0.653), absolute bench press 1RM (*F*_(1,35)_ = 62.944, *p* = 0.000, *η* ^2^ = 0.643), and relative bench press 1RM/kg BW (*F*_(1,35)_ = 80.756, *p* = 0.000, *η* ^2^ = 0.698).

Post hoc pairwise comparisons demonstrated significant within‐group improvements in muscular strength following the 12‐week intervention. In the PRO group, participants showed significant increases in all four strength parameters—absolute and relative 1RM for both the leg press and bench press—relative to baseline (all *p* < 0.01). Moreover, the PRO group exhibited significantly greater gains in absolute leg press 1RM compared to the PLA group (*p* < 0.05). The CT group also showed significant enhancements in all muscular strength outcomes from baseline, including absolute and relative 1RM for both exercises (all *p* < 0.01). In comparison to the PLA group, the CT group demonstrated significantly greater improvements in absolute leg press 1RM (*p* < 0.01), relative leg press 1RM/kg BW (*p* < 0.01), absolute bench press 1RM (*p* < 0.05), and relative bench press 1RM/kg BW (*p* < 0.05).

**FIGURE 3:**
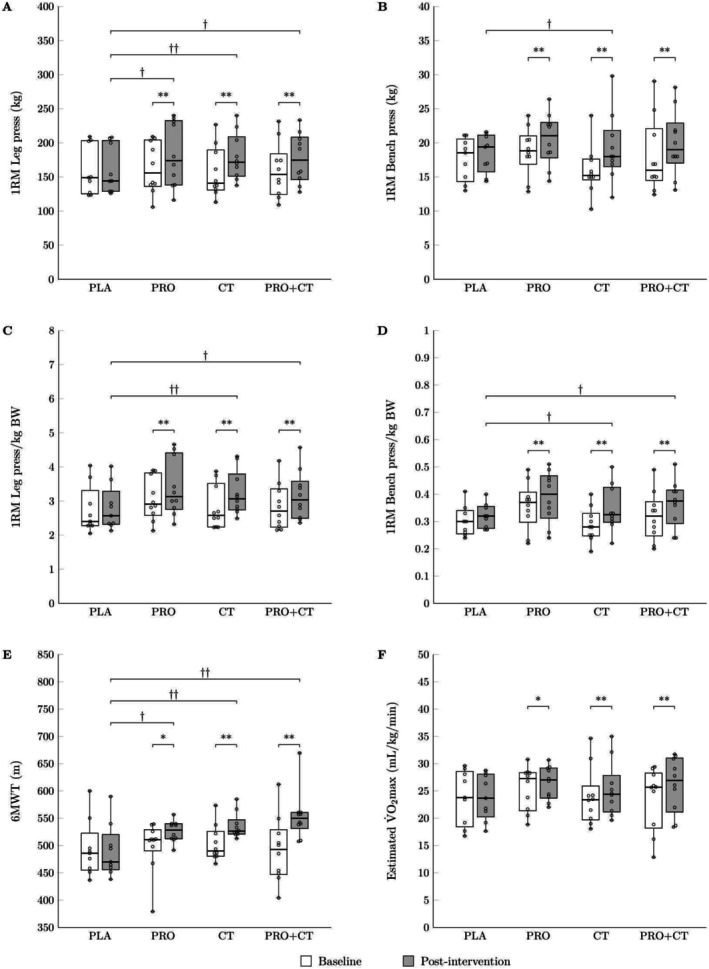
Boxplots illustrating (A) absolute leg press 1RM, (B) absolute bench press 1RM, (C) relative leg press 1RM/kg BW, (D) relative bench press 1RM/kg BW, (E) six‐minute walk test (6MWT) distance, and (F) estimated V̇O_2_max at baseline and after the 12‐week intervention across the study groups: PLA (*n* = 9), PRO (*n* = 10), CT (*n* = 10), and PRO + CT (*n* = 10). Boxes represent the interquartile range (25th–75th percentiles), horizontal lines indicate medians, and whiskers denote minimum and maximum values. PLA, no exercise with placebo supplementation; PRO, no exercise with hydrolyzed house cricket protein supplementation; CT, concurrent training with placebo supplementation; PRO+CT, concurrent training with hydrolyzed house cricket protein supplementation. 1RM, one‐repetition maximum; BW, body weight; V̇O_2_max, maximal oxygen consumption. *p < 0.05 and **p < 0.01 indicate within‐group differences; ^†^ *p* < 0.05 and ^††^ *p* < 0.01 indicate between‐group differences versus PLA.

Participants in the PRO+CT group exhibited the most comprehensive improvements, with statistically significant increases in all four strength metrics—absolute and relative leg press and bench press 1RM—following the intervention (all *p* < 0.01). Additionally, compared to the PLA group, the PRO+CT group achieved significantly greater gains in absolute leg press 1RM (*p* < 0.05), relative leg press 1RM/kg BW (*p* < 0.05), and relative bench press 1RM/kg BW (*p* < 0.05), as illustrated in Figure 3A–D.

### 3.3 Cardiorespiratory Fitness

A two‐way ANOVA with large *η* ^*2*^ revealed statistically significant group‐by‐time interactions for both the 6MWT (*F*_(3,35)_ = 6.806, *p* = 0.001, *η* ^2^ = 0.368) and estimated V̇O_2_max (*F*_(3,35)_ = 5.071, *p* = 0.005, *η* ^2^ = 0.303). Analysis of main effects further indicated that time exerted a significant influence on 6MWT performance (*F*_(1,35)_ = 33.226, *p* = 0.000, *η* ^2^ = 0.487) and estimated V̇O_2_max (*F*_(1,35)_ = 30.530, *p* = 0.000, *η* ^2^ = 0.466). Post hoc pairwise comparisons revealed significant within‐group improvements following the 12‐week intervention. In the PRO group, participants exhibited significant increases in both 6MWT distance and estimated V̇O_2_max (*p* < 0.05 vs. baseline), with a significantly greater improvement in 6MWT performance compared to the PLA group (*p* < 0.05). Similarly, the CT group demonstrated significant enhancements in both 6MWT and estimated V̇O_2_max (*p* < 0.01 vs. baseline), with 6MWT improvements significantly exceeding those of the PLA group (*p* < 0.01). Participants in the PRO + CT group achieved the most pronounced gains, with statistically significant improvements in both 6MWT distance and estimated V̇O_2_max relative to baseline (*p* < 0.01). Furthermore, the PRO + CT group exhibited significantly greater improvements in 6MWT performance compared to the PLA group (*p* < 0.01), as illustrated in Figure 3E,F.

### 3.4 Inflammation and Anabolic Markers

A two‐way repeated measures ANOVA revealed statistically significant group‐by‐time interactions for TNF‐α (*F*_(3,35)_ = 9.400, *p* = 0.000, *η* ^2^ = 0.446) and IGF‐1 (*F*_(3,35)_ = 2.918, *p* = 0.048, *η* ^*2*^ = 0.200), indicating differential changes across groups over the course of the intervention. Although the interaction effects for hs‐CRP (*F*_(3,35)_ = 2.379, *p* = 0.086, *η* ^2^ = 0.169) and CK (*F*_(3,35)_ = 4.043, *p* = 0.052, *η* ^2^ = 0.104) did not reach statistical significance, the corresponding effect sizes suggest potentially meaningful group‐level differences. Analysis of main effects further demonstrated that time had a significant influence on several inflammatory and anabolic markers. Specifically, significant changes over time were observed for hs‐CRP (*F*_(1,35)_ = 17.813, *p* = 0.000, *η* ^2^ = 0.337), TNF‐α (*F*_(1,35)_ = 13.960, *p* = 0.001, *η* ^2^ = 0.285), IGF‐1 (*F*_(1,35)_ = 4.278, *p* = 0.046, *η* ^2^ = 0.109), insulin (*F*_(1,35)_ = 13.238, *p* = 0.001, *η* ^2^ = 0.277), HOMA‐IR (*F*_(1,35)_ = 13.132, *p* = 0.001, *η* ^2^ = 0.273), and CK (*F*_(1,35)_ = 5.017, *p* = 0.005, *η* ^2^ = 0.301), suggesting notable overall improvements following the 12‐week intervention.

Post hoc pairwise comparisons revealed significant within‐group changes. In the PRO group, hs‐CRP (*p* < 0.01) and CK (*p* < 0.05) were significantly reduced compared to baseline. A modest, non‐significant reduction in insulin levels was also observed (*p* = 0.06), with no significant between‐group differences detected for these variables. In the CT group, significant reductions were observed in hs‐CRP (*p* < 0.05), TNF‐α (*p* < 0.01), insulin (*p* < 0.05), and HOMA‐IR (*p* < 0.05), alongside significant increases in IGF‐1 (*p* < 0.05). Compared with the PLA group, the CT group exhibited a significantly greater reduction in TNF‐α (*p* < 0.01), while the between‐group difference in IGF‐1 approached significance (*p* = 0.07).

**FIGURE 4:**
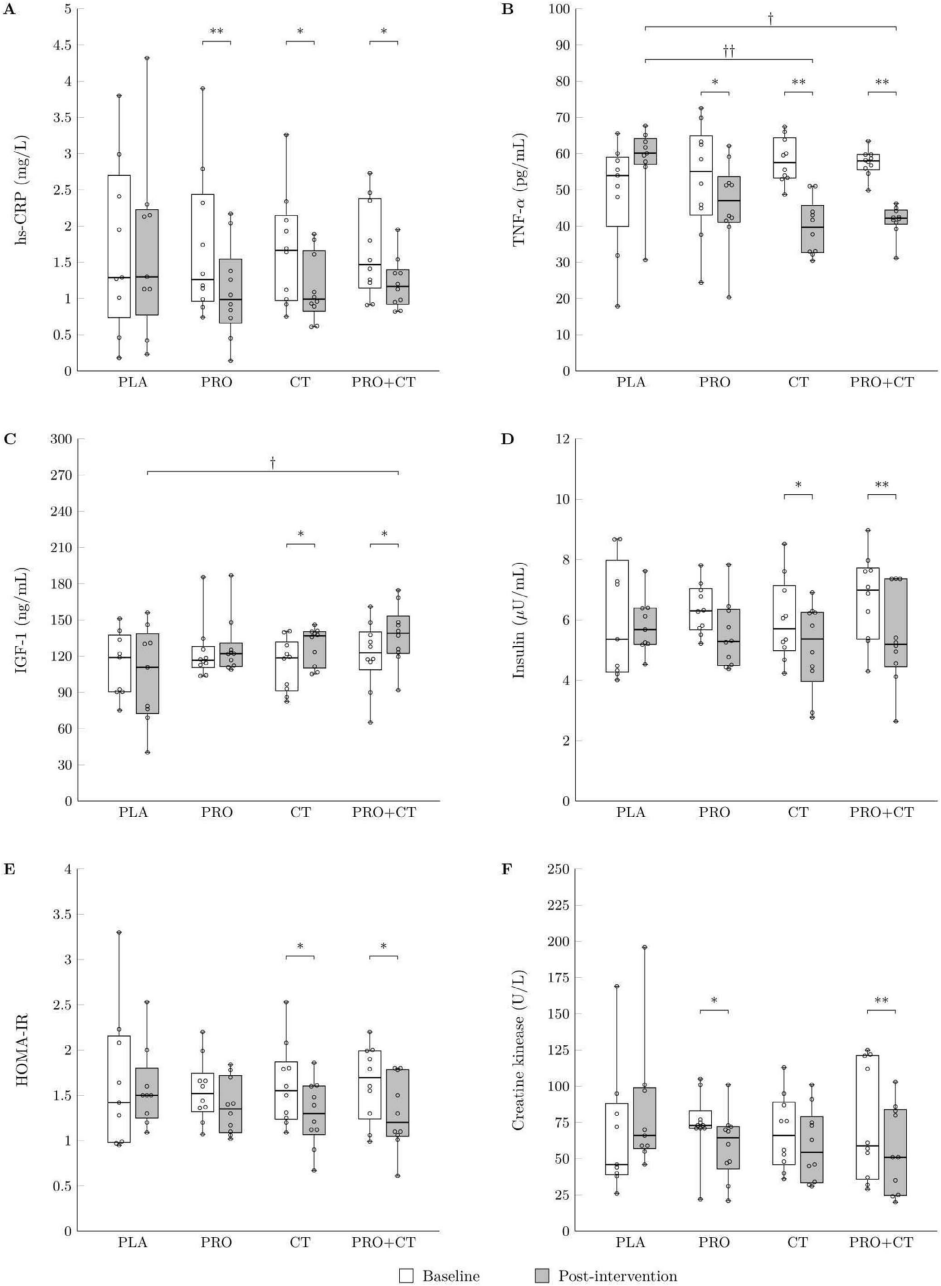
Boxplots illustrating (A) hs‐CRP, (B) TNF‐α, (C) IGF‐1, (D) insulin, (E) HOMA‐IR, and (F) creatine kinase at baseline and after 12 weeks of intervention across the study groups: PLA (*n* = 9), the PRO (*n* = 10), the CT (*n* = 10), and the PRO+CT groups (*n* = 10). Boxes represent the interquartile range (25th–75th percentiles), horizontal lines indicate medians, and whiskers denote minimum and maximum values. PLA, no exercise with placebo supplementation; PRO, no exercise with hydrolyzed house cricket protein supplementation; CT, concurrent training with placebo supplementation; PRO+CT, concurrent training with hydrolyzed house cricket protein supplementation. hs‐CRP, high‐sensitivity C‐reactive protein; TNF‐α, tumor necrosis factor‐alpha; IGF‐1, insulin‐like growth factor 1; HOMA‐IR, homeostasis model assessment of insulin resistance. *p < 0.05 and **p < 0.01 indicate within‐group differences; ^†^ *p* < 0.05 and ^††^ *p* < 0.01 indicate between‐group differences versus PLA.

The PRO+CT group demonstrated the most robust improvements. Significant reductions were observed in hs‐CRP (*p* < 0.05), TNF‐α (*p* < 0.01), insulin (*p* < 0.01), HOMA‐IR (*p* = 0.01), and CK (*p* < 0.01), along with significant increases in IGF‐1 (*p* < 0.05) from baseline. In comparison to the PLA group, the PRO+CT group showed significantly greater reductions in TNF‐α (*p* < 0.05) and significantly greater increases in IGF‐1 (*p* < 0.05), as illustrated in Figure 4.

### 3.5 Metabolic Profiles

Regarding metabolic profile variables, the two‐way repeated measures ANOVA revealed no statistically significant group‐by‐time interactions for FBG, lipid profiles, atherogenic indices, or markers of renal and hepatic function. However, the analysis of main effects indicated that time exerted a significant influence on several outcomes. Specifically, significant time effects were observed for FBG (*F*_(1,35)_ = 3.383, *p* = 0.029, *η* ^2^ = 0.225), TC (*F*_(1,35)_ = 9.825, *p* = 0.003, *η* ^2^ = 0.219), TG (*F*_(1,35)_ = 4.920, *p* = 0.033, *η* ^2^ = 0.123), HDL‐c (*F*_(1,35)_ = 8.174, *p* = 0.007, *η* ^2^ = 0.189), LDL‐c (*F*_(1,35)_ = 14.890, *p* = 0.000, *η* ^2^ = 0.298), AIP (*F*_(1,35)_ = 18.601, *p* = 0.000, *η* ^2^ = 0.347), CRI‐I (*F*_(1,35)_ = 52.456, *p* = 0.000, *η* ^2^ = 0.600), CRI‐II (*F*_(1,35)_ = 43.544, *p* = 0.000, *η* ^2^ = 0.554), and AC (*F*_(1,35)_ = 52.456, *p* = 0.000, *η* ^2^ = 0.600). The results of this study indicate that participants experienced statistically significant improvements in several key indicators of metabolic health over the course of the 12‐week intervention, irrespective of their group allocation.

Post hoc pairwise comparisons revealed significant within‐group improvements following the 12‐week intervention. In the PRO group, participants demonstrated significant reductions in LDL‐c (*p* < 0.05), CRI‐I (*p* < 0.01), CRI‐II (*p* < 0.01), and AC (*p* < 0.01) compared to baseline. The CT group showed significant improvements, including an increase in HDL‐c (*p* < 0.05) and reductions in FBG (*p* < 0.01), CRI‐I (*p* < 0.01), CRI‐II (*p* < 0.01), and AC (*p* < 0.01), with AIP showing a trend toward significance (*p* = 0.05). The PRO+CT group exhibited the most comprehensive metabolic improvements, with significant reductions in TC (*p* < 0.01), TG (*p* < 0.05), LDL‐c (*p* < 0.01), AIP (*p* < 0.01), CRI‐I (*p* < 0.01), CRI‐II (*p* < 0.01), and AC (*p* < 0.01) relative to baseline. However, no significant between‐group differences in metabolic profile variables were observed (Table 3).

**TABLE 3:** Metabolic profiles at baseline and after the 12‐week intervention.

	PLA (*n* = 9)		PRO (*n* = 10)		CT (*n* = 10)		PRO + CT (*n* = 10)		Time effect *η* ^2^ (*p*‐value)	Group × Time interaction *η* ^2^ (*p*‐value)
	Baseline	Post‐test	Baseline	Post‐test	Baseline	Post‐test	Baseline	Post‐test		
FBG (mg/dL)	110.0 ± 33.1	111.8 ± 35.9	98.1 ± 12.1	100.0 ± 6.6	110.2 ± 26.7	102.3 ± 21.1^a^**	97.8 ± 8.8	97.2 ± 8.0	0.225 (0.029)^††,^*	0.019 (0.417)
TC (mg/dL)	186.4 ± 36.3	179.7 ± 32.4	182.1 ± 30.0	163.4 ± 51.6	182.1 ± 31.5	164.0 ± 29.9	204.1 ± 41.5	153.1 ± 52.6^a^**	0.219 (0.003)^††,^**	0.120 (0.210)
TG (mg/dL)	126.9 ± 73.6	105.1 ± 57.4	111.4 ± 58.5	111.2 ± 62.8	91.0 ± 27.6	86.7 ± 26.5	140.0 ± 63.0	101.1 ± 44.3^a^*	0.123 (0.033)^†,^*	0.064 (0.501)
HDL‐C (mg/dL)	41.0 ± 15.1	45.2 ± 13.2	46.4 ± 13.4	52.0 ± 15.2	42.6 ± 10.8	50.4 ± 12.9^a^*	43.8 ± 8.3	46.9 ± 6.6	0.189 (0.007)^††,^**	0.027 (0.812)
LDL‐C (mg/dL)	115.9 ± 29.3	111.9 ± 24.2	109.0 ± 24.7	89.2 ± 38.0^a^*	116.1 ± 27.3	99.6 ± 23.1	128.4 ± 29.1	92.5 ± 38.2^a^**	0.298 (0.000)^††,^**	0.130 (0.177)
AIP	0.45 ± 0.4	0.34 ± 0.3	0.35 ± 0.2	0.28 ± 0.3	0.33 ± 0.2	0.22 ± 0.2	0.47 ± 0.2	0.30 ± 0.2^a^**	0.347 (0.000)^††,^**	0.048 (0.624)
CRI‐I	4.9 ± 1.5	4.3 ± 1.4	4.1 ± 0.8	3.2 ± 0.9^a^**	4.5 ± 1.3	3.4 ± 1.1^a^**	4.7 ± 0.9	3.3 ± 1.2^a^**	0.600 (0.000)^††,^**	0.109 (0.250)
CRI‐II	3.1 ± 1.1	2.7 ± 1.0	2.4 ± 0.6	1.8 ± 0.6^a^**	2.9 ± 1.1	2.1 ± 0.8^a^**	3.0 ± 0.8	2.0 ± 0.8^a^**	0.554 (0.000)^††,^**	0.104 (0.274)
AC	3.9 ± 1.5	3.3 ± 1.4	3.1 ± 0.8	2.2 ± 0.9^a^**	3.5 ± 1.3	2.4 ± 1.0^a^**	3.7 ± 0.9	2.3 ± 1.2^a^**	0.600 (0.000)^††,^**	0.109 (0.250)
BUN (mg/dL)	14.0 ± 5.7	14.5 ± 2.9	16.0 ± 4.4	13.7 ± 2.3	13.4 ± 2.0	13.9 ± 2.5	16.3 ± 2.6	13.9 ± 3.1	0.057 (0.154)	0.117 (0.281)
Creatinine (mg/dL)	0.73 ± 0.1	0.70 ± 0.1	0.72 ± 0.1	0.68 ± 0.1	0.63 ± 0.1	0.62 ± 0.2	0.68 ± 0.1	0.66 ± 0.1	0.050 (0.184)	0.008 (0.963)
AST (U/mL)	13.3 ± 2.9	16.4 ± 4.0	14.2 ± 3.6	16.8 ± 3.9	16.3 ± 2.9	15.8 ± 6.2	15.7 ± 4.5	14.6 ± 4.6	0.043 (0.216)	0.127 (0.184)
ALT (U/mL)	16.6 ± 3.8	17.0 ± 1.9	19.0 ± 5.7	19.5 ± 4.7	18.3 ± 5.0	17.2 ± 3.2	15.9 ± 3.5	14.7 ± 4.7	0.012 (0.514)	0.067 (0.483)

*Note:* Data are presented as mean ± SD. Superscript “†” indicates 0.06 ≤ *η* ^*2*^ < 0.14; Superscript “††” indicates *η* ^2^ ≥ 0.14; * and ** indicate *p* < 0.05 and *p* < 0.01, respectively. Superscript “a” indicates significant differences from baseline within the group.

Abbreviations: AC, atherogenic coefficient; AIP, atherogenic index of plasma; ALT, alanine transaminase; AST, aspartate transaminase; BUN, blood urea nitrogen; CRI‐I, Castelli Risk Index I; CRI‐II, Castelli Risk Index II; CT, concurrent exercise training with placebo supplementation; FBG, fasting blood glucose; HDL‐c, high‐density lipoprotein cholesterol; LDL‐c, low‐density lipoprotein cholesterol; PLA, no exercise training with placebo supplementation; PRO, no exercise training with hydrolyzed house cricket protein supplementation; PRO+CT, concurrent exercise training with hydrolyzed house cricket protein supplementation; TC, total cholesterol; TG, triglycerides.

### 3.6 Quality of Life

A two‐way repeated measures ANOVA revealed statistically significant group‐by‐time interactions for the physical domain (*F*_(3,35)_ = 6.153, *p* = 0.000, *η* ^2^ = 0.345), psychological domain (*F*_(3,35)_ = 8.053, *p* = 0.000, *η* ^2^ = 0.408), social relationships domain (*F*_(3,35)_ = 2.987, *p* = 0.044, *η* ^2^ = 0.204), and overall QoL scores (*F*_(3,35)_ = 15.019, *p* = 0.000, *η* ^2^ = 0.563). No significant group‐by‐time interaction was observed for the environment domain. Analysis of main effects further indicated that time had a statistically significant impact on the physical domain (*F*_(1,35)_ = 27.877, *p* = 0.002, *η* ^2^ = 0.443), psychological domain (*F*_(1,35)_ = 14.932, *p* = 0.000, *η* ^2^ = 0.299), social relationships domain (*F*_(1,35)_ = 6.538, *p* = 0.015, *η* ^2^ = 0.157), and overall QoL scores (*F*_(1,35)_ = 46.755, *p* = 0.000, *η* ^2^ = 0.572). In contrast, no significant main effect of time was found for the environment domain.

Post hoc pairwise comparisons revealed significant within‐group improvements following the 12‐week intervention. In the CT group, participants demonstrated significant increases in the physical domain (*p* < 0.05), psychological domain (*p* < 0.05), and overall QoL scores (*p* < 0.01) relative to baseline. Additionally, the CT group exhibited significantly greater improvements in the psychological domain (*p* < 0.05) compared to the PLA group, while between‐group differences in the physical domain (*p* = 0.06) and overall QoL scores (*p* = 0.05) approached statistical significance.

In the PRO+CT group, participants showed significant improvements across all domains, including the physical domain (*p* < 0.01), psychological domain (*p* < 0.01), social relationships (*p* < 0.01), and overall QoL scores (*p* < 0.01) from baseline. Furthermore, when compared to the PLA group, the PRO+CT group demonstrated significantly greater enhancements in the physical domain (*p* < 0.01), psychological domain (*p* < 0.01), social relationships (*p* < 0.05), and overall QoL scores (*p* < 0.01), as presented in Table 4. In contrast, the PRO group did not exhibit statistically significant changes from baseline in any of the QoL domains, nor were there significant between‐group differences when compared to the PLA group.

**TABLE 4:** Quality of life at baseline and after the 12‐week intervention.

	PLA (*n* = 9)		PRO (*n* = 10)		CT (*n* = 10)		PRO + CT (*n* = 10)		Time effect *η* ^2^ (*p*‐value)	Group × Time interaction *η* ^2^ (*p*‐value)
	Baseline	Post‐test	Baseline	Post‐test	Baseline	Post‐test	Baseline	Post‐test		
Physical domain	29.0 ± 1.2	29.3 ± 0.7	30.4 ± 2.5	30.7 ± 2.2	30.7 ± 2.4	31.3 ± 1.8^a^*	30.7 ± 1.9	32.5 ± 1.3^a^**^,b^**	0.443 (0.000)^††,^**	0.345 (0.002)^††,^**
Psychological domain	25.9 ± 0.8	26.1 ± 0.6	26.2 ± 1.4	25.9 ± 1.5	26.8 ± 1.0	27.5 ± 0.7^a^*^,b^*	26.5 ± 1.1	28.1 ± 0.6^a^**^,b^**	0.299 (0.000)^††,^**	0.408 (0.000)^††,^**
Social relationshops	12.8 ± 0.8	12.8 ± 0.4	13.1 ± 0.6	13.2 ± 0.4	13.0 ± 0.7	13.3 ± 0.5	12.8 ± 0.76	13.4 ± 0.5^a^**^,b^*	0.157 (0.015)^††,^*	0.204 (0.044)^††,^*
Environment	34.0 ± 2.0	34.0 ± 1.7	34.8 ± 1.5	34.8 ± 1.5	33.8 ± 1.9	34.0 ± 2.1	33.6 ± 2.1	33.7 ± 2.1	0.008 (0.607)	0.009 (0.954)
Overall	101.8 ± 1.9	102.2 ± 1.5	104.5 ± 4.5	104.6 ± 4.5	104.3 ± 3.3	106.1 ± 3.0^a^**	103.6 ± 2.9	107.7 ± 2.3^a^**^,b^**	0.572 (0.000)^††,^**	0.563 (0.000)^††,^**

*Note:* Data are presented as mean ± SD. Superscript “††” indicates *η* ^*2*^ ≥ 0.14; * and ** denote *p* < 0.05 and *p* < 0.01, respectively. Superscript “a” indicates significant differences from baseline within the group; “b” indicates significant differences compared to the PLA group at 12 weeks.

Abbreviations: CT, concurrent exercise training with placebo supplementation; PLA, no exercise training with placebo supplementation; PRO, no exercise training with hydrolyzed house cricket protein supplementation; PRO+CT, concurrent exercise training with hydrolyzed house cricket protein supplementation.

## 4 Discussion

To the best of our knowledge, this is the first study to investigate the effects of post‐exercise supplementation with 30 g of hydrolyzed house cricket protein combined with CT on muscle mass, strength, inflammatory markers, and metabolic health related to muscle function in older women. The findings demonstrate that both CT alone and in combination with hydrolyzed cricket protein supplementation (PRO+CT) significantly enhanced muscle mass, muscular strength, key inflammatory and anabolic biomarkers, 6MWT performance, and QoL compared to the placebo group.

Among all interventions, the PRO+CT group demonstrated the most comprehensive benefits, including substantial increases in FFM—particularly in leg LM—marked improvements in upper‐ and lower‐body strength, elevated circulating IGF‐1 levels, and significant reductions in TNF‐α concentrations. These physiological adaptations were accompanied by enhanced CRF and QoL, underscoring the clinical relevance of the intervention in supporting healthy aging in older adults. Collectively, the results suggest a synergistic interaction between exercise‐induced mechanical loading and insect‐derived protein supplementation, offering a promising strategy for enhancing muscular health and overall well‐being in older women.

In terms of body composition, all intervention groups showed significant increases in FFM, ranging from approximately 1.5%–2.0%. Notably, leg LM increased by ~3% in both the PRO and PRO+CT groups. These gains were accompanied by reductions in android fat mass (FM) of approximately 1.5%–3.3%, along with corresponding decreases in the A/G ratio of 2.3%–2.9%. These findings highlight the efficacy of hydrolyzed cricket protein supplementation and CT, both individually and in combination, in promoting skeletal muscle accretion and reducing central adiposity in older women.

Increases in muscle mass were accompanied by significant improvements in muscular strength. Both the CT and PRO+CT groups exhibited marked gains in upper‐ and lower‐body strength, with 1RM values increasing by approximately 16% for the leg press and 20% for the bench press in the CT group, and by 12% for both exercises in the PRO+CT group. These strength improvements are key indicators of functional capacity and independence in older adults. The PRO group also showed a significant increase in lower‐body strength (11.2% in leg press 1RM), but no improvement in upper‐body strength, suggesting that cricket protein supplementation may preferentially enhance frequently used muscle groups, such as the lower limbs. However, in the absence of mechanical loading, upper‐body strength gains may be limited.

In the CT and PRO+CT groups, the exercise intervention likely incorporated both AT and resistance‐based movements targeting the upper and lower limbs. The RT component imposed substantial mechanical loading on large muscle groups—particularly those of the lower extremities—while also engaging upper‐body musculature essential for daily functional activities. As a result, these combined modalities are expected to elicit robust hypertrophic and strength adaptations across multiple regions of the body. Additionally, the inclusion of AT in the CT protocol likely contributed to the observed reductions in central adiposity, as reflected by significant decreases in android fat mass and the A/G ratio. These findings are consistent with a recent systematic review and meta‐analysis demonstrating that CT is comparably effective to AT in reducing body fat and equally effective to RT in promoting muscle mass gains among middle‐aged and older adults (Khalafi et al. 2025). This aligns with evidence indicating that CT enhances muscular strength through increased motor unit recruitment, muscle hypertrophy, and improved metabolic efficiency (Chen et al. 2024). Therefore, the integration of AT and RT within the CT framework may exert synergistic effects that optimize both musculoskeletal and metabolic adaptations in older adults.

The observed synergistic effect may be attributed to the high‐quality amino acid profile of the hydrolyzed cricket protein, which comprised 76.96% protein and 57.00 mg/100 g of free amino acids. Among the most abundant were glutamic acid, aspartic acid, alanine, leucine, valine, and isoleucine. Notably, leucine, valine, and isoleucine play key roles in muscle protein metabolism. Leucine is a potent stimulator of muscle protein synthesis (MPS) through activation of the mechanistic target of rapamycin (mTOR) signaling pathway, while valine and isoleucine support muscle energy metabolism by serving as oxidative substrates, enhancing glucose uptake, and promoting tissue repair during and after exercise (Kaspy et al. 2023). These effects may contribute to the observed reductions in CK levels in both the PRO and PRO+CT groups, potentially through modulation of muscle damage markers and facilitation of muscle recovery. Notably, recent evidence suggests that cricket protein and whey protein elicit comparable activation of the mTOR pathway (Lanng et al. 2023), indicating that insect‐derived proteins may be similarly effective in stimulating MPS when consumed post‐exercise (Jackman et al. 2017). Additionally, glutamic and aspartic acids may facilitate recovery and immune regulation through their roles in nitrogen transport and neurotransmitter synthesis. Alanine, a glucogenic amino acid, may further aid energy homeostasis during prolonged physical activity (Miyajima 2020). Collectively, this unique amino acid composition likely contributed to the enhanced anabolic and anti‐inflammatory responses observed with cricket protein supplementation combined with CT.

In the PRO group, the greater improvements in lower‐limb strength may be attributed to the inherent functional demands placed on these muscles during daily activities such as walking, standing, and postural transitions. These routine movements likely provided sufficient mechanical stimulus to elicit region‐specific anabolic responses when combined with protein supplementation. This suggests that lower‐limb musculature may be particularly responsive to nutritional support, even in the absence of structured exercise, highlighting the role of habitual physical activity in modulating musculoskeletal adaptations in older adults. Notably, hydrolyzed house cricket protein supplementation not only supported overall gains in muscle mass but also appeared to preferentially enhance lower‐body functional outcomes—key for maintaining mobility, balance, and independence in aging populations (McGregor et al. 2014).

Importantly, the gains in muscle mass and strength observed in the PRO+CT group were accompanied by favorable changes in key inflammatory and anabolic biomarkers. Significant reductions in circulating TNF‐α, along with increased IGF‐1 levels, suggest that the combined intervention not only attenuated systemic inflammation but also enhanced the anabolic environment conducive to MPS and hypertrophy. These biochemical adaptations likely contributed to the increases in FFM—particularly leg LM—and improvements in muscular strength. While CT alone also reduced TNF‐α levels, the addition of hydrolyzed cricket protein appeared to amplify this response, indicating a synergistic effect between exercise‐induced mechanical stimuli and nutritional supplementation.

The reductions in TNF‐α observed in both the CT and PRO+CT groups are particularly noteworthy, given the central role of this pro‐inflammatory cytokine in the development of sarcopenia and age‐related functional decline (Draganidis et al. 2016). In this study, AT—a key component of the CT protocol—likely contributed to the anti‐inflammatory effects. Previous research indicates that AT suppresses pro‐inflammatory cytokine production by reducing macrophage infiltration into skeletal muscle and downregulating nuclear factor‐kappa B, a key regulator of inflammation (Cavalcante et al. 2017; Lesniewski et al. 2011). Notably, increases in circulating IGF‐1 concentrations were observed exclusively in the PRO+CT group, highlighting the distinct efficacy of combining hydrolyzed cricket protein supplementation with CT. IGF‐1 is a key anabolic hormone that regulates skeletal muscle remodeling by stimulating myoblast proliferation and promoting MSP through activation of the PI3K/Akt/mTOR signaling pathway—critical for muscle hypertrophy and regeneration (Yoshida and Delafontaine 2020).

The synergistic effect observed in the PRO+CT group likely reflects the complementary mechanisms of the intervention components: while the AT and RT elements of CT reduce pro‐inflammatory cytokines such as TNF‐α, the amino acid‐rich cricket protein may have enhanced anabolic signaling by stimulating IGF‐1 production (Solerte et al. 2008). By concurrently reducing inflammation and enhancing anabolic signaling, the PRO+CT intervention appears to address both catabolic and anabolic processes underlying age‐related muscle loss (Dalle et al. 2017). This dual‐action mechanism likely accounts for the superior improvements in muscle mass and strength observed in the PRO+CT group, emphasizing the clinical potential of combining functional exercise with high‐quality, insect‐derived protein supplementation to support musculoskeletal health in aging populations.

In contrast, the PRO group showed no significant between‐group differences in inflammatory or anabolic biomarkers compared to the PLA group, despite modest within‐group improvements. This highlights the essential role of exercise‐induced mechanical and metabolic stimuli in amplifying the physiological effects of protein supplementation. The absence of significant changes without structured training suggests that nutrient‐driven adaptations—such as enhanced MSP and anti‐inflammatory responses—are most effectively achieved when protein intake is temporally aligned with physical activity. These findings underscore the synergistic interaction between exercise and nutrition, indicating that the anabolic and anti‐inflammatory benefits of hydrolyzed cricket protein supplementation are maximized when combined with CT.

Regarding metabolic profiles, the absence of significant group‐by‐time interactions indicates that, although modest within‐group improvements were noted, the effects did not differ meaningfully across intervention groups. These findings underscore the challenge of modifying metabolic risk factors in older adults and suggest that longer intervention periods, greater training volumes, or more potent nutritional strategies may be necessary to achieve significant between‐group differences in metabolic outcomes.

Significant improvements in CRF, as assessed by the 6MWT, were observed in the PRO, CT, and PRO+CT groups. These findings support the efficacy of the PRO+CT in enhancing aerobic capacity, consistent with prior meta‐analyses in older adults (Lin et al. 2021; Yoshimura et al. 2025). The improvements highlight the established role of AT in promoting CRF and underscore the added benefits of integrating AT and RT within the CT framework. AT induces central cardiovascular adaptations—such as enhanced oxygen delivery and utilization—as well as peripheral changes, including increased skeletal muscle capillarization, which collectively improve aerobic performance (Hurst et al. 2019). The RT component likely contributed to gains in muscular strength and endurance, supporting locomotor efficiency and sustained submaximal effort during the 6MWT. These enhancements in functional endurance are clinically meaningful, as they reflect a key aspect of physical performance in aging populations. The superior outcomes observed in the PRO+CT group suggest a synergistic interaction between exercise‐induced mechanical loading and hydrolyzed cricket protein supplementation, fostering optimal physiological conditions for improving both central cardiovascular function and peripheral muscle efficiency in older adults.

Improvements in muscle strength and CRF were accompanied by significant increases in QoL scores, particularly within the physical, psychological, and social domains. Both the CT and PRO+CT groups demonstrated notable enhancements in the physical domain of QoL, likely driven by gains in upper‐ and lower‐limb strength—key contributors to functional independence in daily activities such as standing, walking, and stair climbing. Increases in muscle mass and strength may have also reduced perceptions of fatigue, frailty, and physical limitations commonly associated with sarcopenia and aging.

Beyond physical benefits, the CT and PRO+CT groups exhibited significant improvements in psychological QoL, reflecting enhanced emotional well‐being, life satisfaction, and mental health. These findings align with existing evidence linking regular physical activity to reduced symptoms of anxiety and depression in older adults (Rahmati et al. 2024). Additionally, cricket protein supplementation may have contributed to improved recovery, reduced muscle damage, and attenuated physical stress, further enhancing the psychological benefits of training (Alhebshi et al. 2021).

Importantly, the PRO+CT group also showed significant improvements in social QoL, highlighting the broader impact of the combined intervention on social engagement and perceived support. The observed multidimensional QoL enhancements support the notion that integrating hydrolyzed cricket protein supplementation with structured CT yields superior outcomes compared to either intervention alone. Collectively, these findings emphasize the value of a comprehensive, integrative approach to counteracting age‐related physical and psychological decline, thereby promoting overall well‐being and functional independence in older adults.

This double‐blind, randomized, placebo‐controlled trial demonstrated high adherence and a low dropout rate; however, several limitations should be acknowledged. The relatively small sample size may have limited the statistical power to detect subtle between‐group differences. Additionally, the study population consisted primarily of older women, which limits the generalizability of the findings, as sex‐based physiological differences may influence responses to exercise and protein supplementation. The 12‐week intervention period may have been insufficient to elicit significant changes in lipid profiles and atherogenic indices, suggesting that longer follow‐up durations are warranted to assess the long‐term effects. Although adherence to protein supplementation in the PRO group was reportedly high and monitored via self‐reported intake logs, the lack of objective dietary validation limits the ability to attribute observed effects solely to the supplementation. Participants were instructed to maintain their habitual dietary patterns, and no significant differences in daily intake were reported among groups.

Future studies should include larger, sex‐stratified cohorts to enhance generalizability and explore potential sex‐specific responses. Moreover, comparative trials evaluating cricket protein against established protein sources are recommended to further assess its anabolic efficacy and viability as a sustainable nutritional strategy for aging populations.

## 5 Conclusions

This study demonstrates that combining hydrolyzed house cricket protein supplementation with CT elicits superior benefits in muscle mass, muscular strength, inflammatory modulation, and anabolic signaling, alongside improvements in CRF and QoL. These findings highlight a synergistic interaction between insect‐derived protein and structured exercise in enhancing muscle mass, strength, and functional capacity among older women. Moreover, the results underscore the potential of cricket protein as a sustainable and effective nutritional strategy to augment the impact of exercise‐based interventions aimed at mitigating sarcopenia and preserving physical independence in aging populations.

## Author Contributions

**Pilanee Vaithanomsat:** conceptualization (equal), funding acquisition (lead), project administration (equal), resources (lead), writing – original draft (supporting). **Udomlak Sukatta:** investigation (equal), methodology (equal). **Phornpimon Janchai:** investigation (equal), methodology (equal). **Piyaporn Tumnark:** data curation (equal), formal analysis (equal). **Jatuporn Phoemsapthawee:** conceptualization (lead), data curation (equal), formal analysis (equal), investigation (equal), methodology (equal), project administration (equal), writing – original draft (lead), writing – review and editing (lead).

## Conflicts of Interest

The authors declare no conflicts of interest.

## Supporting information


**Table S1:** Composition and amino acid profile of hydrolyzed house cricket ( *A. domesticus* ) protein powder.
**Table S2:** Total daily energy intake and macronutrient composition at baseline and during the intervention across all groups.

## Data Availability

The data that supports the findings of this study is available in Tables S1 and S2 of this article.
