# Supplementary material for: Enhancing Muscle Strength and Reducing Inflammation: The Combined Effects of Hydrolyzed House Cricket Protein Supplementation and Concurrent Training in Older Women
Source: Food Sci Nutr. 2025 Sep 22;13(9):e70984. doi: 10.1002/fsn3.70984 (PMC12454676; doi:10.1002/fsn3.70984)
Supplement: Supplementary file 1 — Table S1: Composition and amino acid profile of hydrolyzed house cricket ( A. domesticus ) protein powder. Table S2: Total daily energy intake and macronutrient composition at baseline and during the intervention across all groups. [file FSN3-13-e70984-s001.docx]

**Table S1.** Composition and amino acid profile of hydrolyzed house cricket (*A. domesticus*) protein powder

| **Hydrolysate Powder** | **Composition** |
| --- | --- |
| **Protein (%)** | 76.96 |
| **Total Amino Acids** (mg/100g) |  |
| Alanine | 5.08 |
| Arginine | 3.72 |
| Aspartic acid | 7.17 |
| Glutamic acid | 9.20 |
| Glycine | 2.82 |
| Histidine | 1.21 |
| Isoleucine | 2.17 |
| Leucine | 4.37 |
| Lysine | 3.73 |
| Phenylalanine | 1.92 |
| Proline | 3.19 |
| Serine | 3.88 |
| Threonine | 2.49 |
| Tyrosine | 2.13 |
| Valine | 3.92 |
| **Carbohydrates (%)** | 4.53 |
| **Fat (%)** | 0.55 |
| **Dietary fiber (%)** | 9.48 |
| **Ash (%)** | 6.75 |
| **Moisture (%)** | 1.73 |

**Table S2.** Total daily energy intake and macronutrient composition at baseline and during the intervention across all groups.

|  | **PLA (*n* = 9)** | | **PRO (*n* = 10)** | | **CT (*n* = 10)** | | **PRO+CT (*n* = 10)** | | **Time effect**  ***η*^2^ (*P*-value)** | **Group×Time interaction**  ***η*^2^ (*p*-value)** |
| --- | --- | --- | --- | --- | --- | --- | --- | --- | --- | --- |
|  | **Baseline** | **Post-test** | **Baseline** | **Post-test** | **Baseline** | **Post-test** | **Baseline** | **Post-test** |  |  |
| Energy intake (Kcal/day) | 1812.3 ± 135.2 | 1840.1 ± 143.4 | 1835.6 ± 184.2 | 1821.8 ± 137.4 | 1776.3 ± 106.3 | 1806.1 ± 81.7 | 1758.7 ± 110.2 | 1795.4 ± 102.7 | 0.060 (0.143) | 0.060 (0.530) |
| Energy intake (Kcal/kg BW/day) | 30.9 ± 1.1 | 31.1 ± 1.3 | 33.8 ± 3.3 | 33.9 ± 3.9 | 31.9 ± 1.9 | 32.5 ± 2.3 | 31.4 ± 2.9 | 32.0 ± 3.3 | 0.063 (0.134) | 0.030 (0.778) |
| Protein (g) | 67.5 ± 6.5 | 67.7 ± 5.9 | 62.2 ± 8.9 | 63.3 ± 7.2 | 64.7 ± 3.0 | 65.0 ± 4.6 | 61.2 ± 6.0 | 62.7 ± 5.9 | 0.066 (0.126) | 0.042 (0.677) |
| Protein (g/kg BW/day) | 1.2 ± 0.1 | 1.2 ± 0.1 | 1.2 ± 0.1 | 1.2 ± 0.1 | 1.2 ± 0.1 | 1.2 ± 0.1 | 1.1 ± 0.1 | 1.1 ± 0.0 | 0.034 (0.276) | 0.040 (0.693) |
| Protein (Kcal) | 269.8 ± 25.9 | 270.9 ± 23.9 | 250.5 ± 35.6 | 253.1 ± 29.0 | 258.9 ± 12.1 | 259.8 ± 18.2 | 244.7 ± 24.0 | 250.7 ± 23.7 | 0.069 (0.117) | 0.043 (0.671) |
| Protein (E %) | 14.9 ± 0.9 | 14.7 ± 0.9 | 14.1 ± 1.2 | 14.0 ± 1.2 | 14.6 ± 0.6 | 14.4 ± 0.7 | 14.0 ± 0.7 | 14.0 ± 1.1 | 0.000 (0.952) | 0.089 (0.348) |
| CHO (g) | 232.1 ± 29.8 | 233.8 ± 24.8 | 229.1 ± 25.8 | 233.9 ± 19.3 | 221.9 ± 24.1 | 223.5 ± 18.0 | 218.0 ± 23.5 | 218.5 ± 11.0 | 0.022 (0.385) | 0.011 (0.941) |
| CHO (g/kg BW/day) | 4.0 ± 0.4 | 4.1 ± 0.4 | 4.2 ± 0.6 | 4.3 ± 0.5 | 4.0 ± 0.4 | 4.0 ± 0.4 | 3.9 ± 0.5 | 3.9 ± 0.3 | 0.026 (0.337) | 0.020 (0.870) |
| CHO (Kcal) | 928.2 ± 119.3 | 935.9 ± 99.5 | 916.5 ± 103.1 | 935.5 ± 77.2 | 887.5 ± 96.4 | 894.0 ± 71.9 | 871.8 ± 94.2 | 878.1 ± 44.6 | 0.029 (0.315) | 0.009 (0.958) |
| CHO (E %) | 51.2 ± 5.3 | 50.9 ± 4.7 | 50.0 ± 4.8 | 51.0 ± 4.1 | 50.0 ± 4.4 | 50.0 ± 3.5 | 49.5 ± 3.2 | 49.7 ± 2.4 | 0.001 (0.854) | 0.029 (0.791) |
| Fat (g) | 68.3 ± 11.2 | 70.4 ± 12.1 | 74.3 ± 15.5 | 70.4 ± 14.6 | 70.0 ± 10.2 | 72.5 ± 7.9 | 71.4 ± 5.3 | 73.9 ± 10.3 | 0.010 (0.562) | 0.104 (0.271) |
| Fat (g/kg BW/day) | 1.2 ± 0.2 | 1.2 ± 0.2 | 1.3 ± 0.3 | 1.3 ± 0.3 | 1.3 ± 0.2 | 1.3 ± 0.1 | 1.3 ± 0.1 | 1.3 ± 0.2 | 0.031 (0.298) | 0.097 (0.307) |
| Fat (Kcal) | 614.2 ± 100.8 | 633.3 ± 108.4 | 638.7 ± 139.3 | 633.1 ± 131.5 | 630.0 ± 91.6 | 652.3 ± 70.6 | 641.2 ± 48.1 | 635.5 ± 92.7 | 0.010 (0.561) | 0.105 (0.269) |
| Fat (E %) | 33.9 ± 5.0 | 34.3 ± 4.5 | 36.3 ± 5.7 | 34.6 ± 5.4 | 35.5 ± 4.6 | 36.1 ± 3.6 | 36.6 ± 3.0 | 36.2 ± 5.7 | 0.000 (0.973) | 0.081 (0.394) |

**Note:** Data are shown as means ± SD. PLA, no exercise training with placebo supplementation; PRO, no exercise training with hydrolyzed house cricket protein supplementation; CT, concurrent exercise training with placebo supplementation; PRO+CT, concurrent exercise training with hydrolyzed house cricket protein supplementation; BW, body weight; CHO, carbohydrate.
